# Lah is a transmembrane protein and requires Spa10 for stable positioning of Woronin bodies at the septal pore of *Aspergillus fumigatus*

**DOI:** 10.1038/srep44179

**Published:** 2017-03-10

**Authors:** Yannik Leonhardt, Sara Carina Kakoschke, Johannes Wagener, Frank Ebel

**Affiliations:** 1 Max-von-Pettenkofer-Institute, Ludwig-Maximilians-University, Munich, 80336, Germany; 2 Institute for Infectious Diseases and Zoonoses, Ludwig-Maximilians-University, Munich, 80539, Germany

## Abstract

Woronin bodies are specialized, fungal-specific organelles that enable an immediate closure of septal pores after injury to protect hyphae from excessive cytoplasmic bleeding. In most Ascomycetes, Woronin bodies are tethered at the septal pore by so-called Lah proteins. Using the pathogenic mold *Aspergillus fumigatus* as a model organism, we show that the C-terminal 288 amino acids of Lah (LahC_288_) bind to the rim of the septal pore. LahC_288_ essentially consists of a membrane spanning region and a putative extracellular domain, which are both required for the targeting to the septum. In an *A. fumigatus rho4* deletion mutant that has a severe defect in septum formation, LahC_288_ is recruited to spot-like structures in or at the lateral membrane. This suggests that LahC is recruited before Rho4 starts to govern the septation process. Accordingly, we found that in wild type hyphae Lah is bound before a cross-wall emerges and thus enables a tethering of Woronin bodies at the site of the newly formed septum. Finally, we identified Spa10, a member of a recently described family of septal pore-associated proteins, as a first protein that directly or indirectly interacts with LahC to allow a stable positioning of Woronin bodies at the mature septum.

Eukaryotic cells have a complex composition comprising different organelles and many other specialized substructures. To function properly these parts of the cellular machinery have to be arranged in a certain spatial pattern. The precise positioning of organelles therefore represents an important element of the cellular architecture. A fascinating example of such a positioning is the anchoring of Woronin bodies to the cell envelope of filamentous fungi. Two distinct spatial patterns have evolved: In the *Neurospora/Sordaria* clade, Woronin bodies are tethered to the lateral cell wall^1^ whereas in most other Ascomycetes they are positioned directly at the septal pore^2^ Woronin bodies protect hyphae from extensive cytoplasmic bleeding after damage. They have been shown to be important for the stress resistance and virulence of the plant pathogen *Magnaporthe grisea*^3^ the nematophagous fungus *Arthrobotrys oligospora*^4^ and the human pathogenic mold *A. fumigatus*^5^. In the latter, a lack of Woronin bodies furthermore results in an enhanced sensitivity for the cell wall-damaging antimycotic agent caspofungin^6^.

Woronin bodies are fungal-specific organelles that derive from specialized peroxisomes^7^ and contain two characteristic components. The so-called Hex protein assembles into a paracrystalline structure that fills the matrix of the Woronin body^8^. The other characteristic constituent is a membrane protein, designated WSC in *N. crassa* and WscA in *A. fumigatus*. Both proteins have been shown to be essential for the biogenesis of Woronin bodies, but they apparently differ with respect to their roles in the anchoring of Woronin bodies. WSC was shown to interact with the N-terminus of the leashin protein LAH-1^1^ whereas such a direct involvement in the tethering process is unlikely for WscA^9^.

The lateral binding pattern of Woronin bodies found in *Neurospora* is characteristic for a small group of fungi. It most likely developed from an ancestral pattern found in most other Ascomycetes to meet the specific requirements of fungi that grow unusually fast^10^. It is assumed that due to the characteristic cytoplasmic streaming towards the apical tips of *Neurospora*, septum-associated Woronin bodies would immediately block the septal pores. In most other Ascomycetes, Woronin bodies are positioned in close proximity to the septal pore and if activated can immediately close this connection to neighboring cells^2^. For this group of fungi, *Aspergillus* Lah is the prototypic leashin protein^5^,^11^.

In eukaryotic cells, the positioning of organelles is often mediated by interactions with the cytoskeleton, which provides a physical frame work that spans the whole cell^12^,^13^. The positioning of Woronin bodies seems to be independent of cytoskeletal elements and instead relies on giant tethering proteins of approximately 500.000 Da. These proteins have been designated leashins to emphasize their common function that, however, is not always based on sequence homology. The leashin LAH-1 found in the *Neurospora/Sordaria* clade shows no homology to the Lah proteins of most other Ascomycetes^1^,^5^ but both types of leashins link Woronin bodies either to the hyphal envelope or the septal pore. Thus, two distinct tethering proteins determine the two positioning patterns of Woronin bodies found in filamentous fungi.

We have recently shown that the N-terminal domain of the leashin Lah binds to the surface of Woronin bodies, whereas its C-terminal part is targeted to the rim of the septal pore^5^. The LahC domain that was initially described comprised 1000 amino acids (aa), which corresponds to a molecular weight of more than 110 kDa. In this study, we have narrowed down the LahC targeting domain to 288 aa. Moreover, we provide evidence that this domain harbors a membrane-spanning region that is essential for its function. Finally, we have identified Spa10 as the first septum-associated protein that is required for a stable anchoring of Woronin bodies at the septal pore.

## Results

### Sequence analysis of the C-terminal 1000 amino acids of Lah

**Figure 1:**
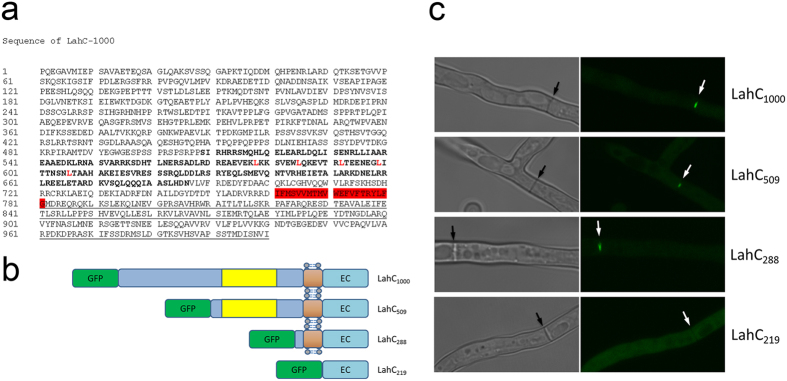
Identification of the minimal LahC domain. Panel (a) Sequence analysis of the C-terminal 1000 aa of Lah. The putative coiled-coil region is indicated in bold. The leucine residues of a putative leucine zipper are indicated in red. The predicted transmembrane region is highlighted in red. A region that is also conserved in *N. crassa* LAH-2, is underlined. Panel (b and c) GFP-LahC_1000_ and several derivatives thereof containing truncated versions of LahC were generated. These constructs are schematically depicted in panel (b). The putative coiled-coil (yellow), transmembrane (brown) and extracellular domains (EC) are indicated. Panel (c) shows the localization of the different fusion proteins in *A. fumigatus* hyphae. Each image represents a projection of a stack of confocal images covering hypha in their whole depth. Arrows indicate the positions of septa.

We have recently shown that the C-terminal 1000 aa of Lah are sufficient to target GFP to the central part of the septal cell wall^5^. In this study, we have further analysed the corresponding amino acid sequence (Fig. 1a). The SMART program predicts a coiled-coil domain comprising residues 509–686 including several leucine residues that may form a leucine zipper (as predicted by the 2ZIP–Server). A putative inside-to-outside transmembrane region was predicted for residues 761–781 by TMpred and PHOBIUS. Remarkably, we found that the transmembrane region is also conserved in *N. crassa* LAH-2 as well as in the Lah proteins of *A. nidulans* (FGSC A4), *A. oryzae* (RIB40), *A. terreus* (NIH2624) and *A. flavus* (NRRL 3357) (data not shown).

### A domain consisting of 288 amino acid targets Lah to the septal pore

Starting from GFP-LahC_1000_ we constructed a series of fusion proteins comprising smaller parts of the C-terminus of Lah (Fig. 1b). Fusions containing the C-terminal 509 (GFP-LahC_509_) or 288 aa (GFP-LahC_288_) showed the same targeting to the septum as GFP-LahC_1000_ (Fig. 1c). LahC_288_ lacks the putative coiled-coil domain, but comprises the predicted transmembrane region (Fig. 1b). GFP-LahC_219_, which lacks this putative membrane-spanning segment, was not recruited to the septum, but evenly spread in the cytosol instead (Fig. 1b and c). This demonstrates that the putative transmembrane sequence is required for the correct targeting of LahC.

**Figure 2:**
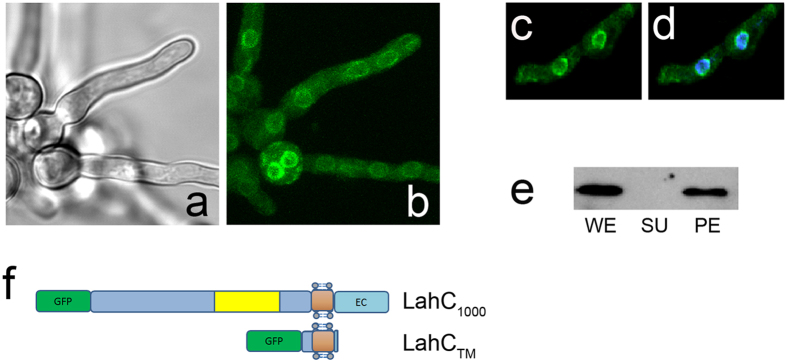
GFP-LahC_TM_ is a membrane protein and localizes to perinuclear organelle-like structures. GFP-LahC_1000_ and the GFP-LahC_TM_ construct are schematically depicted in panel (f). The putative coiled-coil (yellow), transmembrane (brown) and extracellular domains (EC) are indicated. GFP-LahC_TM,_ localizes in circular structures in the cytoplasm of *A. fumigatus* (panel b). Panel (a) shows a corresponding brightfield image. Panel (c) shows GFP-LahC_TM_ fluorescence and panel (d) the corresponding overlay of GFP-LahC_TM_ and DAPI fluorescence. Panel (e) shows an anti-GFP immunoblot of whole cell extract (WE), supernatant and pellet fraction obtained after ultracentrifugation (SU and PE, respectively).

In the next step, we generated another truncated derivative of GFP-LahC_288_ lacking the putative extracellular domain. This fusion protein, designated GFP-LahC_TM_, comprises the membrane spanning region and short stretches of flanking residues. As shown in Fig. 2b, it is not recruited to the septum, but enriched in circular, organelle-like structures in the cytoplasm. A parallel DAPI staining revealed a perinuclear pattern for this GFP fusion protein (Fig. 2c and d). These microscopic images already suggested that GFP-LahC_TM_ is a membrane protein. To verify this, lysates of *A. fumigatus* expressing GFP-LahC_TM_ were fractionated by ultracentrifugation and subsequently analysed by immunoblot. A GFP-fusion protein of the expected size was detectable in the whole cell lysate and in the pellet fraction, but not in the supernatant (Fig. 2e), which supports the notion that LahC_TM_ is a membrane protein. To provide further evidence, we introduced two point mutations resulting in two amino acid substitutions (V_5303_→E_5303_ and V_5304_→E_5304_) that eliminated the predicted transmembrane sequence (Supplementary Fig. S1). The corresponding fusion protein, designated GFP-LahC_288_*, is not targeted to the septal pore (Supplementary Fig. S1) demonstrating that the membrane-spanning segment is essentially required for a correct localization of LahC.

### LahC and Woronin bodies are recruited at an early time point during septation

**Figure 3:**
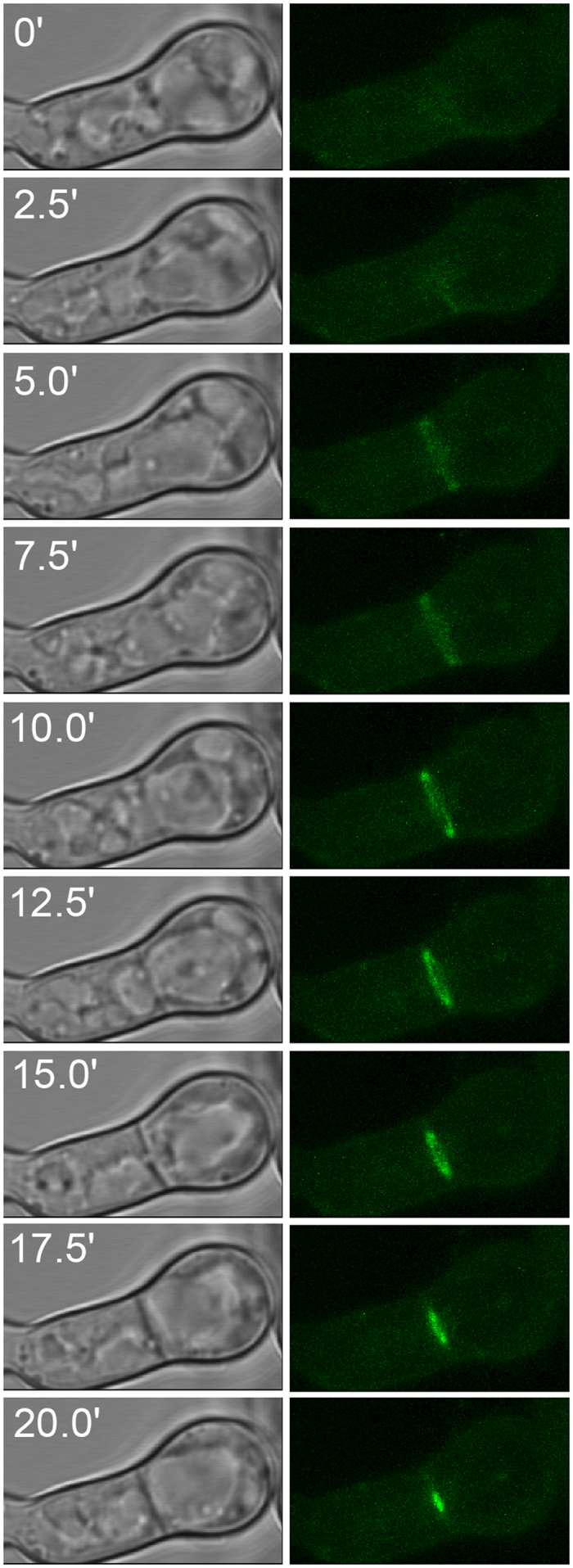
Recruitment of LahC during septum formation. Live cell imaging of an *A. fumigatus* strain expressing GFP-LahC_288_. Images are projections of stacks of confocal images that were taken in intervals of 2.5 min.

**Figure 4:**
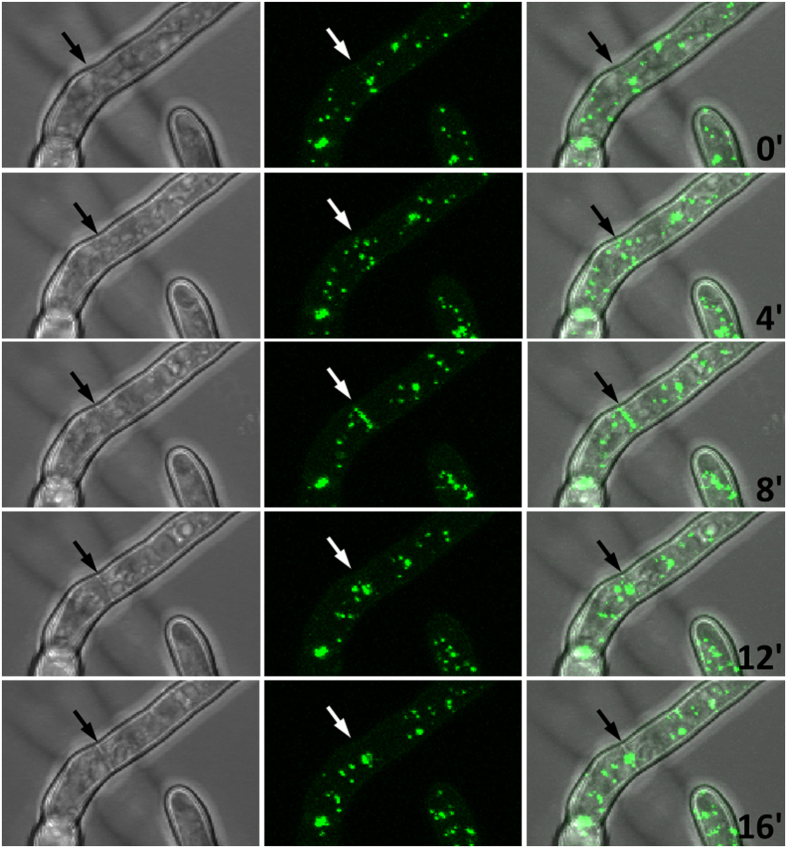
Recruitment of Woronin bodies during septum formation. Panels show *A. fumigatus* hyphae expressing LahN-GFP to visualize Woronin bodies. The micrographs are projections of stacks of confocal images that were taken every 4 min. Arrows indicate the position of an emerging septum.

The Lah protein tethers Woronin bodies to the rim of the septal pore, but it was unknown whether LahC is recruited at an early or late stage of the septation process. In order to address this question, we analyzed hyphae by live cell microscopy. Figure 3 shows a hyphal segment with a newly formed septum. The images clearly show the formation and constriction of a GFP-LahC ring during septum formation. Strikingly, LahC is recruited even before a cross wall is visible (after 5 and 7.5 min). In a strain expressing GFP-LahC_288_ and LifeAct-RFP, LahC appeared at the site of septation approximately at same time as the contractile actin ring (Supplementary Fig. S2). The early recruitment of LahC suggested that Woronin bodies may already been tethered at the sites of emerging septa. To address this point we generated an *A. fumigatus* wild type strain in which Woronin bodies are visualized by expression of LahN-GFP. As shown in Fig. 4, Woronin bodies indeed gather in the plane of the forming septum, before they are finally arranged in the characteristic cluster proximal to the septal pore. We also analysed the septation process in an *A. fumigatus* strain co-expressing LahN-GFP and LifeAct-RFP. A set of images is shown in the Supplementary Fig. 3. As soon as the newly formed septum became visible by bright-field microscopy, Woronin bodies appeared to be tethered at the emerging cross wall, whereas the formation of the contractile actin ring was evident 3 min earlier (Supplementary Fig. S3).

**Figure 5:**
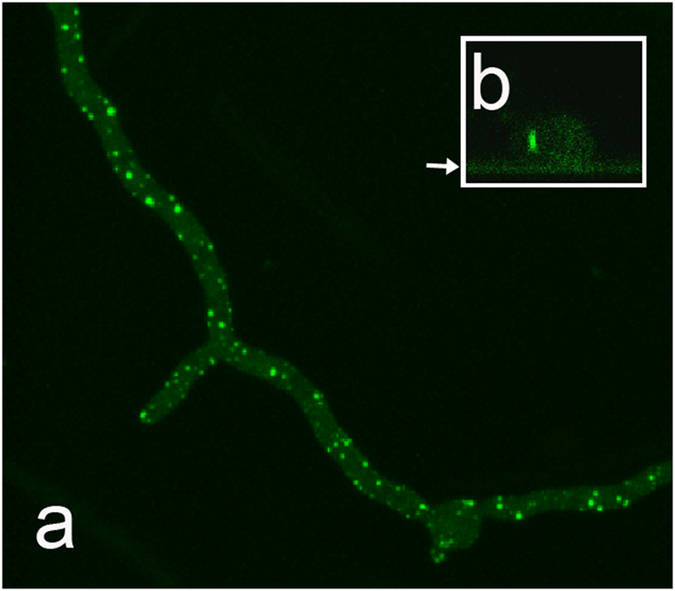
Localization of GFP-LahC_288_ in the Δ*rho*4 mutant. Panel (a) shows the localization of GFP-LahC_288_ in distinct spots. Panel (b) shows xz-cross sections of a hypha demonstrating that the GFP-LahC_288_-positive spots localize at the lateral membrane. The position of the cover slip is indicated by an arrow.

We have recently shown that an *A. fumigatus* ∆*rho*4 mutant has a severe defect in septum formation^14^ Expression of GFP-LahC_288_ in this mutant led to a striking localization pattern. GFP-LahC_288_ was strongly enriched in spot-like structures (Fig. 5a) and cross-sections by confocal xz analysis showed that these structures localize in or at the lateral membrane (Fig. 5b). Live cell imaging revealed that these structures are stable and show a limited motility in the plane of the cytoplasmic membrane (Video 1). Live cell microscopy of a ∆*rho*4 mutant strain expressing GFP-HexA revealed that some GFP-tagged Woronin bodies were clearly associated with the lateral wall for longer times (Supplementary Fig. S4), a pattern that was never observed in the wild type (data not shown). In conclusion, our data suggest that in the absence of Rho4 certain protein complexes assemble in or at the cytoplasmic membrane that recruit GFP-LahC_288_ and can thereby tether Woronin bodies.

### Analysis of the role of Spa10 during septum formation

The data described so far suggest that the LahC receptor is a membrane protein with an extracellular domain that interacts with the extracellular domain of LahC. The putative LahC receptor is recruited early to the site of septation and shows a stable localization at the rim of the mature septal pore. Literature searches revealed a candidate for this receptor with a promising localization pattern, namely Spa10, which has briefly been characterized in *A. nidulans*^15^. Its *A. fumigatus* counterpart, encoded by Afu4g13320, is predicted to possess a membrane-spanning region with a high probability for an N-terminus-out configuration. We therefore constructed a Spa10-RFP fusion protein and expressed it in *A. fumigatus.* This fusion protein indeed localized to the septal cross-wall, but unlike LahC, this localization appeared to be less focussed around the septal pore (Supplementary Fig. S5). During septum formation in the wild type, Spa10-RFP appeared, when a septal cross wall was already visible by light microscopy (Supplementary Fig. S6). In the Δ*rho*4 mutant, Spa10-RFP was not recruited to discrete spot-like structures like GFP-LahC_288_, but localized along the lateral membrane instead, a pattern that was particular prominent in conidial bodies (Supplementary Fig. S5). A closer inspection of wild type hyphae revealed a similar, but less prominent localization at the lateral cell wall (data not shown). The clearly distinct localisation patterns of Spa10-RFP and GFP-LahC in the Δ*rho*4 mutant argued against a direct interaction of these proteins. However, to investigate the role of Spa10 in the septation process in more detail, we generated an *A. fumigatus spa*10 deletion mutant. The generation and verification of this deletion is schematically depicted in Supplementary Fig. S7. The mutant showed no obvious defect in growth compared to wild type, both at 37 °C or 50 °C. In the presence of the cell wall stressor Calcofluor white the mutant grew like wild type and showed only a slight delay in conidiation (Supplementary Fig. S7). Analysis of septum formation by Calcofluor white staining revealed also no obvious defects for the mutant (data not shown).

**Figure 6:**
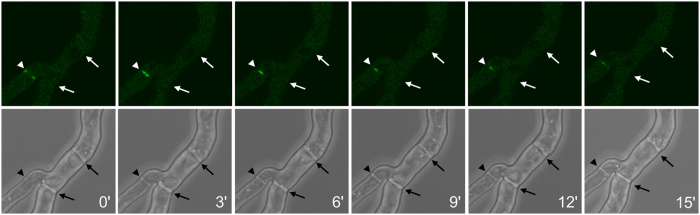
Live cell imaging of GFP-LahC_288_ in the Δ*spa*10 mutant. Images represent projections of stacks of confocal images and were taken at 3 min intervals. Arrows indicate the positions of septa. The arrowheads indicate the position of a newly formed septum.

A striking phenotype of the Δ*spa*10 mutant became apparent after expression of GFP-LahC_288_. Initially, we found no distinct localization of GFP-LahC_288_ in this mutant, but a closer inspection revealed a striking recruitment of GFP-LahC_288_ to the sites of newly formed septa (Fig. 6). As in wild type hyphae, GFP-LahC is recruited early, when a cross wall was hardly visible, and during the centripetal growth of the septal wall its localization became more and more focused to the center of the septation plane. Unlike wild type, the GFP-LahC-positive structures at the mature septum of the mutant lost their fluorescence over time. After 15 min these structures were hardly detectable and at later time points many of them disappeared completely (Fig. 6 and data not shown).

**Figure 7:**
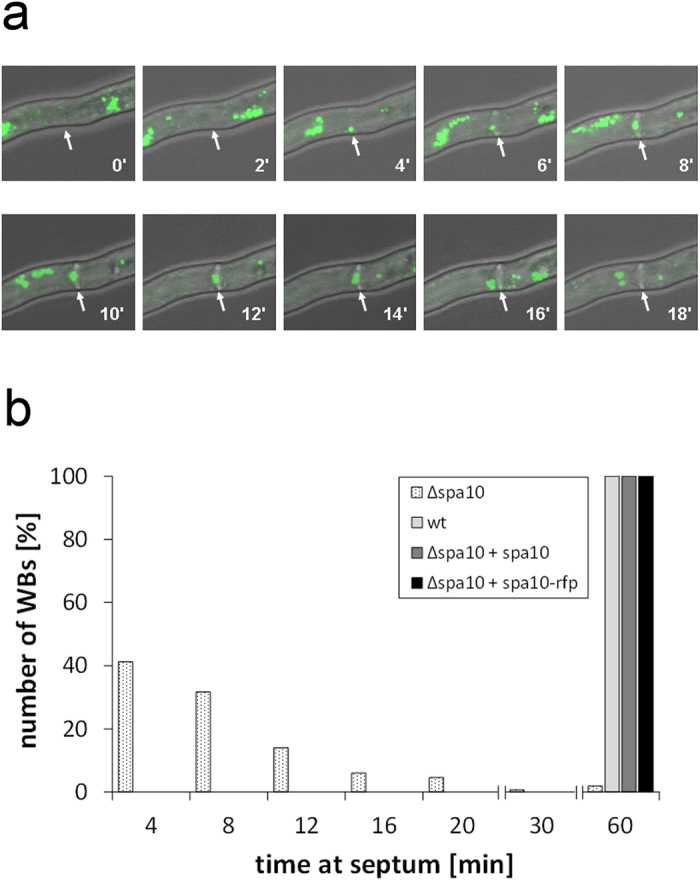
In the absence of Spa10, Woronin bodies are not or only transiently tethered to the septum. The micrographs in panel (a) show a hypha of the Δ*spa*10 mutant expressing GFP-HexA. The position of an emerging septum is indicated by arrows. Images represent projections of stacks of confocal images that were taken every 2 min as indicated. Panel (b) shows a quantification of the time Woronin bodies remained at the septal pore. These data derived from live cell imaging of the indicated strains (30 septa per strain).

**Figure 8:**
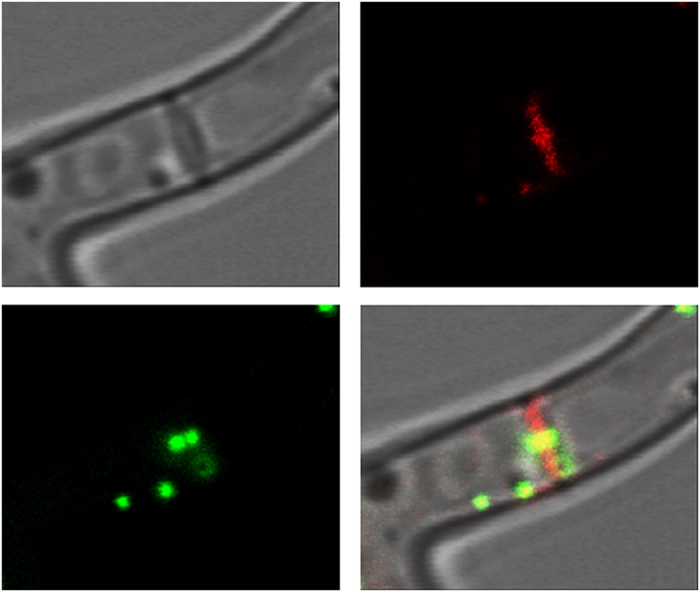
Complementation of the Δ*spa*10 mutant by expression of Spa10-RFP. The localization of Spa10-RFP and Woronin bodies tagged with GFP-HexA is shown in red and green, respectively.

We hypothesized that the dramatically reduced amounts of LahC found at the mature septa of the ∆*spa*10 mutant should have consequences for the positioning of Woronin bodies. To prove this, we expressed GFP-HexA in this mutant. In the resulting strain, only very few Woronin bodies were found in proximity to a septum and live cell microscopy revealed that these associations were only accidental or transient (Fig. 7a). Expression of Spa10 or Spa10-RFP in the mutant restored the wild type phenotype with Woronin bodies anchored at either side of the septum (Fig. 8 and data not shown). Spa10-RFP was enriched in the vicinity of the septal pore, but in contrast to GFP-LahC_288_, also found at other parts of the septal cross wall (Fig. 8).

Using live cell imaging, we also determined the average time a Woronin body remained at the septal pore (Fig. 7b). These experiments were run for up to 60 min. All Woronin bodies in the wild type and the complemented Δ*spa*10 mutant (*spa*10 or *spa*10-*rfp*) remained at the septum for the whole time period, only the Δ*spa*10 mutant showed a striking defect with an average time of 9.0 min. Another feature of this mutant that distinguishes it from the wild type is the tendency of Woronin bodies to form clusters (Fig. 7a), a pattern resembling that found in a mutant expressing a truncated Lah protein, which lacks its C-terminal domain^5^. In conclusion, our data demonstrate that in the absence of Spa10, LahC is unable to reside at the mature septal pore and consequently Woronin bodies cannot or only transiently associate with the septum.

## Discussion

The precise positioning of a protein usually requires a specific interaction with a binding partner that is often part of a larger structural framework. Protein domains usually mediate these interactions and represent key elements of the positioning machinery. The Lah protein tethers Woronin bodies at the septum and its C-terminal domain is specifically targeted to the rim of the septal pore^5^. In this study, we have defined a minimal binding domain of 288 aa that is well conserved in different *Aspergillus* Lah proteins and *N. crassa* LAH-2. A coiled-coil domain, predicted for positions 512 to 685 of LahC_1000_, is not required for the targeting to the septal pore. It may, however, be involved in protein-protein interactions between individual Lah proteins, which have been described for *A. fumigatus* and *A. oryzae*^5^,^11^. In the targeting domain, a transmembrane region is predicted with high probability that divides LahC_288_ in a small intracellular part of 49 aa and a larger extracellular domain of 219 residues. In a septum-bound Lah protein the latter domain would extend into the extracellular space that is formed by the cytoplasmic membrane, which encloses the septal cross-wall. A GFP fusion comprising only the extracellular domain showed no distinct localisation demonstrating that the putative transmembrane region is required for the interaction with the unknown binding partner of Lah at the septum.

A GFP fusion comprising the putative transmembrane region and very short stretches of flanking residues (LahC_TM_) showed a distinct localization, but not at the septal pore. Instead it was enriched in the membrane of round organelle-like structures that enclose nuclei. The localization of LahC_TM_ is very similar to that observed for the *A. fumigatus* NCE102 homologue, which resides in the Endoplasmatic Reticulum^16^. We therefore assume that LahC_TM_ is recruited to the nuclear domain of the Endoplasmatic Reticulum^17^. We initially expected Lah to be a solely cytoplasmic linker between Woronin bodies and the rim of the septal pore. The predicted transmembrane region and the localization of LahC_TM_ challenged this notion. Two lines of evidence furthermore suggest that the C-terminal domain of Lah harbors a trans-membrane region, which is essential for the function of LahC: (i) in protein extracts separated by ultracentrifugation. LahC is found in the pellet fraction and (ii) elimination of the predicted transmembrane region by introduction of point mutations abrogates the targeting of LahC to the septal pore.

It was shown previously that the vegetative hyphae of an *A. fumigatus* Δ*rho4* mutant contains no or only very few septa^14^. In non-septate hyphae, we expected GFP-LahC_288_ to be diffusively distributed in the cytoplasmic membrane. Surprisingly, it showed a clearly distinct localization pattern and was targeted to distinct spot-like structures in or at the cytoplasmic membrane. Live cell analysis revealed a minor lateral mobility for these structures. We assume that these spots represent protein complexes consisting of components of the early septation machinery. They assemble in a Rho4-independent manner, but at a later stage, when Rho4 activity becomes indispensable, the septation process comes to a halt in the Δ*rho4* mutant. Si *et al*.^18^ have shown for *A. nidulans* that Rho4 is required for the formation of the contractile actin ring (CAR). We therefore compared the temporal recruitment of LahC and actin using a strain that co-expresses GFP-LahC and LifeAct-RFP and observed an approximately simultaneous recruitment. Thus, Rho4 may control the assembly of the CAR, but not the simultaneous recruitment of LahC. It was an unexpected finding that LahC is already recruited at such an early step of the septation process. However, additional data confirmed that Lah proteins are recruited at this stage, since we observed Woronin bodies that are apparently tethered to nascent septa. According to our current knowledge, it is unlikely that Woronin bodies are directly involved in the septation process, and we therefore assume that the putative LahC receptor represents an essential constituent of the nascent septum, which most likely serves other functions apart from Lah binding.

It is remarkable that the LahC-recruiting spot-like structures persist in the cytoplasmic membrane of the *rho*4 mutant for longer times. Their ability to bind substantial amounts of LahC suggested that some Woronin bodies may be linked to the lateral cell wall of this mutant. Indeed we observed Woronin bodies at this position and thus in a pattern similar to that found in *N. crassa*.

At this stage, we assumed that the receptor of LahC is recruited early during septation and resides permanently at the mature septal pore. Moreover, we hypothesized that it is a membrane protein with an extracellular domain that interacts with the extracellular domain of LahC. Potential candidates might be found in the Spa-family of septal pore-associated proteins^19^. Some of them have already been analysed in *A. nidulans*^15^. Based on these localization data and predictions of transmembrane regions, Spa10 appeared to be a particular promising candidate. A Spa10-RFP fusion expressed in *A. fumigatus* was indeed found to be targeted to the septum. During septum formation, Spa10-RFP appears later than LahC, at a stage when a cross-wall is already visible. Moreover, in comparison to LahC, Spa10-RFP seems to be less focussed at the centre of the septal cross-wall. It was additionally detectable in the cytoplasmic membrane of *A. fumigatus*, a localization that is particularly prominent in conidial bodies and that was never observed for LahC. Thus, there is a clear difference in the temporal and spatial patterns of LahC and Spa10-RFP, which argues against a direct interaction of these proteins. In order to investigate the role of Spa10 in the process of septation, we deleted the corresponding gene. The Δ*spa*10 mutant showed no dramatic defects in growth, colony morphology or sporulation. However, targeting of GFP-LahC_288_ to the mature septum was nearly completely abolished in this mutant and only rarely we observed very weak spots of GFP-LahC_288_ in the centre of septal cross-walls. However, during septum formation the situation was strikingly different. At this stage, GFP-LahC_288_ was strongly recruited; it formed a large ring that constricted parallel to the centripetal growth of the cross wall. At the mature septum, GFP-LahC_288_ signals gradually disappeared and the mutant showed a severe defect in the anchoring of Woronin bodies at the septal pore. In conclusion, these data indicate that Spa10 is not required for the initial recruitment of LahC during septum formation, but it is essential for a stable localization of LahC at the septal pore.

In the Δ*rho*4 mutant, Spa10-RFP is apparently not recruited to the GFP-LahC_288_-containing spots. If these structures are indeed remnants of halted septation events, they should contain only those proteins that are recruited in the early steps of the septation process. The absence of Spa10-RFP demonstrates that Spa10 is recruited at a later stage and in a Rho4-dependent manner. Since Spa10 is dispensable for the early recruitment of LahC, we assume that a so far unknown protein or protein complex attracts LahC to the site of septation.

**Figure 9:**
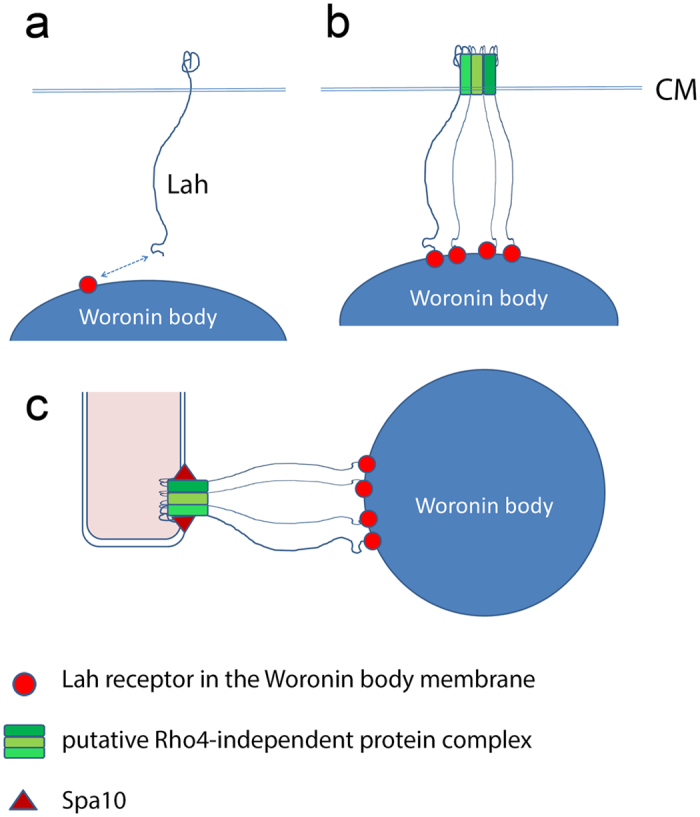
Hypothetical model of the events leading to the recruitment of Lah and Woronin bodies during septum formation. (**a**) Lah is a transmembrane protein which resides with its C-terminal part in the cytoplasmic membrane. As long as this protein is diffusely spread in the membrane, its interactions with Woronin bodies are most likely weak and transient. (**b**) We propose that very early during the septation process a putative protein complex assembles in a Rho4-independent manner and recruits Lah proteins via their C-terminal domains. At this stage, Woronin bodies may already be tethered by bundles of Lah proteins. (**c**) In the mature septum, Spa10 is required for a stable anchoring of Woronin bodies. We assume that Spa10 stabilizes the already formed LahC-recruiting protein complex and thereby enables a stable positioning of Woronin bodies.

In conclusion, this study provides new insights in the architecture and function of Lah. We show that the C-terminal 288 amino acids are sufficient to recruit Lah to the septum. Our data suggest a sequence of events that is depicted in Fig. 9. Newly formed Lah molecules insert with their C-terminal part in the cytoplasmic membrane, while their N-termini are free to interact with their receptor on the Woronin body surface (Fig. 9a). Given that the anchoring of a Woronin body requires a tethering by many Lah molecules, it is likely that interactions mediated by single or only few Lah molecules are weak and only transient. Thus, at this stage the Lah molecules, which are dispersed in the cytoplasmic membrane, are most likely unable to tether Woronin bodies. When the septation process starts, an early protein complex assembles in a Rho4-independent manner that efficiently recruits Lah. This allows multiple interactions involving many Lah proteins, which already enable an early tethering of Woronin bodies (Fig. 9b). Interestingly, this stage seems to be somehow conserved in the lateral spot-like structures found in the Δ*rho*4 mutant. When the mature septal cross wall is finally formed, a stable localization of LahC at the septal pore requires Spa10 (Fig. 9c). Spa10 may either directly interact with LahC or, more likely, it is stabilizing a larger protein complex that interacts with LahC. In any case, our study identifies Spa10 as the first molecule that is essentially required for a stable positioning of Woronin bodies at the septal pore. To achieve a stable anchoring of a Woronin body at a defined position of the cell envelope, interactions with components of the cell wall are most likely required. Thus, protein complexes may exist in the cytoplasmic membrane that provide docking sites for the two types of leashins and additionally interact with the cell wall. The Spa10 protein may be a first component of such a putative protein complex, but further research is clearly required to define these structures in more detail.

## Methods

### Strains and media

The *A. fumigatus* strain AfS35 is a derivative of strain D141 lacking the homologous end-joining component AkuA and the ∆rho4 mutant have been described previously^14^,^20^. Aspergillus minimal medium (AMM) was prepared and resting conidia were isolated as described in^5^,^21^ respectively.

### Sequence analysis and data base searches

Sequences from different Aspergillus species were obtained from the Aspergillus genome database (AspGD; http://www.aspergillusgenome.org/). Homology searches were performed using BlastP at Fungal Genomes Central - NCBI - NIH (http://www.ncbi.nlm.nih.gov/projects/genome/guide/fungi/). For sequence alignments we used Clustal Omega (http://www.ebi.ac.uk/Tools/msa/clustalo/). Transmembrane regions were predicted using TMpred (http://www.ch.embnet.org/software/TMPRED_form.html) or PHOBIUS (http://phobius.sbc.su.se/). Further analysis of protein sequences was performed using SMART (http://smart.embl.de/) and 2ZIP - Server (http://2zip.molgen.mpg.de/cgi-bin/2zip.pl).

### Construction of strains expressing fluorescent fusion proteins

The LifeAct-RFP plasmid was a kind gift of Dr. W.J. Steinbach (Duke University Medical Center, Durham, NC, USA)^22^. The GFP-HexA, GFP-LahC_1000_ and LahN-GFP constructs have been described previously^5^,^23^.

To generate the different truncated LahC derivatives schematically depicted in Figs 2 and 3, suitable fragments were amplified by PCR using the Q5 high fidelity polymerase (New England Biolabs) and the oligonucleotides given in Table 1. The resulting blunt end PCR products were subsequently cloned into the EcoRV site of pSK379sGFP. To generate the Spa10-RFP construct, the *spa10* sequence was amplified by PCR (without STOP codon) and cloned into the PmeI site of pSK379mRFP. To generate the complementation construct, the *spa10* sequence was amplified by PCR using primer combination Spa10-fwd/Spa10-stop-rev and cloned into the PmeI site of pSK379. All construct were sequenced before transformation.

### Generation of point mutations

The transmembrane region at the C terminus of Lah was mutated using the Change-IT™ Multiple Mutation Site Directed Mutagenesis Kit (Affymetrix) and the oligonucleotide ‘LahC-mutation’ according to the instructions of the vendor. The mutations were verified by sequencing.

### Analysis of protein extracts

Aspergillus minimal medium (AMM) was inoculated with conidia of *A. fumigatus* AfS35 strain expressing GFP-LahC_TM_ and the culture was incubated with shaking at 37 °C for 16 h. The resulting mycelium was harvested and washed in 10 mM HEPES buffer (pH 7.4). The pellet was resuspended in HEPES buffer and a lysate was generated using a FastPrep24 (MP Biomedicals). The extract was cleared by centrifugation (15 min, 10,000 g) and subsequently fractionated by ultracentrifugation (100,000 g for 60 min). Samples representing similar fractions of the initial extract were separated by SDS-PAGE electrophoresis and blotted onto a nitrocellulose membrane. The blot was finally developed using a rabbit anti-GFP antibody and a suitable peroxidase-labeled secondary antibody.

### Microscopy and live cell imaging

To analyse fluorescent fusion proteins, conidia of the respective strains were inoculated in 8-well ibidi-chambers or 60 μ-dishes (ibidi GmbH, Martinsried, Germany) containing AMM. Germ tubes were generated by overnight incubation at 30 °C. Fungal cells were then grown to the desired length and the samples were analysed using a Leica SP-5 microscope equipped with an environmental chamber adjusted to 37 °C (Leica Microsystems). To analyse fluorescent fusion proteins, conidia of the respective strains were inoculated in 8-well ibidi-chambers or 60 μ-dishes (ibidi GmbH) containing AMM. When the hyphae reached an appropriate length, they were analysed using a Leica SP-5 microscope equipped with an environmental chamber adjusted to 37 °C (Leica Microsystems, Wetzlar, Germay). All micrographs were taken using a Leica HCX PL APO lamda blue 63 × 1.4 Oil UV objective. Further image processing was performed using Adobe Photoshop CS. Time-lapse confocal images were recorded using a Leica SP-5 microscope under the conditions described above. The resulting image stacks were merged and exported as avi files using the SP-5 and ImageJ software. To quantify the time that a Woronin body remained at a certain septum, strains expressing GFP-HexA were analysed by live cell imaging. 30 septa per strain were analysed by generating image stacks every 2 min over a period of 1 h.

### Construction of the Δspa10 mutant strain

All oligonucleotides used to generate the deletion and complementation constructs are listed in Table 1. For cloning experiments, PCR reactions were performed using the Q5 high fidelity polymerase (New England Biolabs). To construct a suitable replacement cassette, a 3.5 kb hygromycin resistance cassette was excised from pSK346 using SfiI. 1000 bp upstream and downstream of the *spa*10 gene (Afu4g13320) were amplified by PCR from chromosomal DNA using the oligonucleotide pairs ∆spa10-5′-fwd/∆spa-5′-rev and ∆spa-3′-fwd/∆spa-3′-rev. After digestion with SfiI the PCR products were ligated to the hygromycin cassette. The resulting deletion construct was transformed into protoplast of *A. fumigatus* strain AfS35 and the cells were then transferred to AMM plates containing 1.2 M sorbitol and either 200 μg/ml hygromycin (Roche, Applied Science, Mannheim, Germany). To verify the mutant, we amplified *spa*10 using oligonucleotides spa10-fwd/spa10-rev (PCR1). The inserted hygromycin cassette was detected using the primer combinations ∆spa10-5′-cast/gpdA(p)-3-rev (PCR2) and hph-3-SmaI/∆spa10-3′-cast (PCR3). The complementation of the mutant with *spa*10 and *spa*10-*rfp* was verified using the primer combinations gpdA(p)-3-seq-fwd/Spa10-rev and gpdA(p)-3-seq-fwd/seq-mRFP1-rev.

### Phenotypic plate assays

Isolated conidia were counted using a Neubauer chamber. For drop dilution assays, a series of ten-fold dilutions starting with 5 × 10^7^ conidia/ml were spotted onto AMM plates in aliquots of 3 μl; if indicated, plates were supplemented with 25 μg/ml Calcofluor white and incubated at 37 °C.

## Additional Information

**How to cite this article:** Leonhardt, Y. *et al*. Lah is a transmembrane protein and requires Spa10 for stable positioning of Woronin bodies at the septal pore of *Aspergillus fumigatus. Sci. Rep.* **7**, 44179; doi: 10.1038/srep44179 (2017).

**Publisher's note:** Springer Nature remains neutral with regard to jurisdictional claims in published maps and institutional affiliations.

## Supplementary Material

Supplementary Video 1

Supplementary Information

## Figures and Tables

**Table 1:** Oligonucleotides used in this study.

Designation	Sequence	Restriction site
LahC1044-fwd	GCG**TGTACA**GCCATTGCAGAGTTCGACGAG	Bsp1407I
LahC509-fwd	GA**TGTACA**GAGGGATGGGGGGAGTCGCCC	Bsp1407I
LahC288-fwd	GA**TGTACA**TTCTCCAAACACTCCGACCAT	Bsp1407I
LahC219-fwd	GA**TGTACA**ATGGATCGTGAGCAACGCCAA	Bsp1407I
LahC-TM-rev	GAGATATCCTACTTGAGCTTTTGGCGTTGCTC	
LahC-rev	TCAGATCACGTTGCTGATATCCATGGTCGA	
LahC-mutation	ATATCTTCATGTCCGAGGAGATGACTATGGTGTG	
Spa10-fwd	ATGGGTGTCGACACCCGCAGG	
Spa10-rev	CGATGGTCGTGTTCAACGTCAACCTGGTC	
Spa10-stop-rev	TCAATGGTCGTGTTCAACGTCAA	
∆spa10-5′-fwd	ATCAACACTAGGTTAACTAGCTAGC	
∆spa10-5′-rev	CG**GGCCATCTAGGCC**CTCGGTAGGAACCAGTGTG	SfiI
∆spa10-3′-fwd	GT**GGCCTGAGTGGCC**TCGGTTGTCACGCAACATC	SfiI
∆spa10-3′-rev	CTACCCTGTTGGAGGGAGAT	
∆spa10-5′-cast	TCTGGGTTGCTGGTCCTCC	
∆spa10-3′-cast	GGATTGGCTTCACTGGCAATGTTAG	
gpdA(p)-3-rev	TGTCTGCTCAAGCGGGGTAG	
XylP-rev	AAACGTTGGTTCTTCGAGTCGATGAATG	
gpdA(p)-3-seq-fwd	ACTCCATCCTTCCCATCCC	
seq-mRFP1-rev	TTCACGGAGCCCTCCATG	
