# Supplementary material for: Lah is a transmembrane protein and requires Spa10 for stable positioning of Woronin bodies at the septal pore of Aspergillus fumigatus
Source: Sci Rep. 2017 Mar 10;7:44179. doi: 10.1038/srep44179 (PMC5345055; doi:10.1038/srep44179)
Supplement: Supplementary Information [file srep44179-s2.pdf]

Supplementary information.

Lah is a transmembrane protein and requires Spa10 for stable positioning of Woronin bodies at the septal pore of *Aspergillus*.

Yannik Leonhardt[1#], Sara Carina Kakoschke[1#], Johannes Wagener[1], Frank Ebel[2,1*]

1: Max-von-Pettenkofer-Institute, Ludwig-Maximilians-University, Munich, 80336, Germany

2: Institute for Infectious Diseases and Zoonoses, Ludwig-Maximilians-University, Munich, 80539, Germany

#: both authors contributed equally

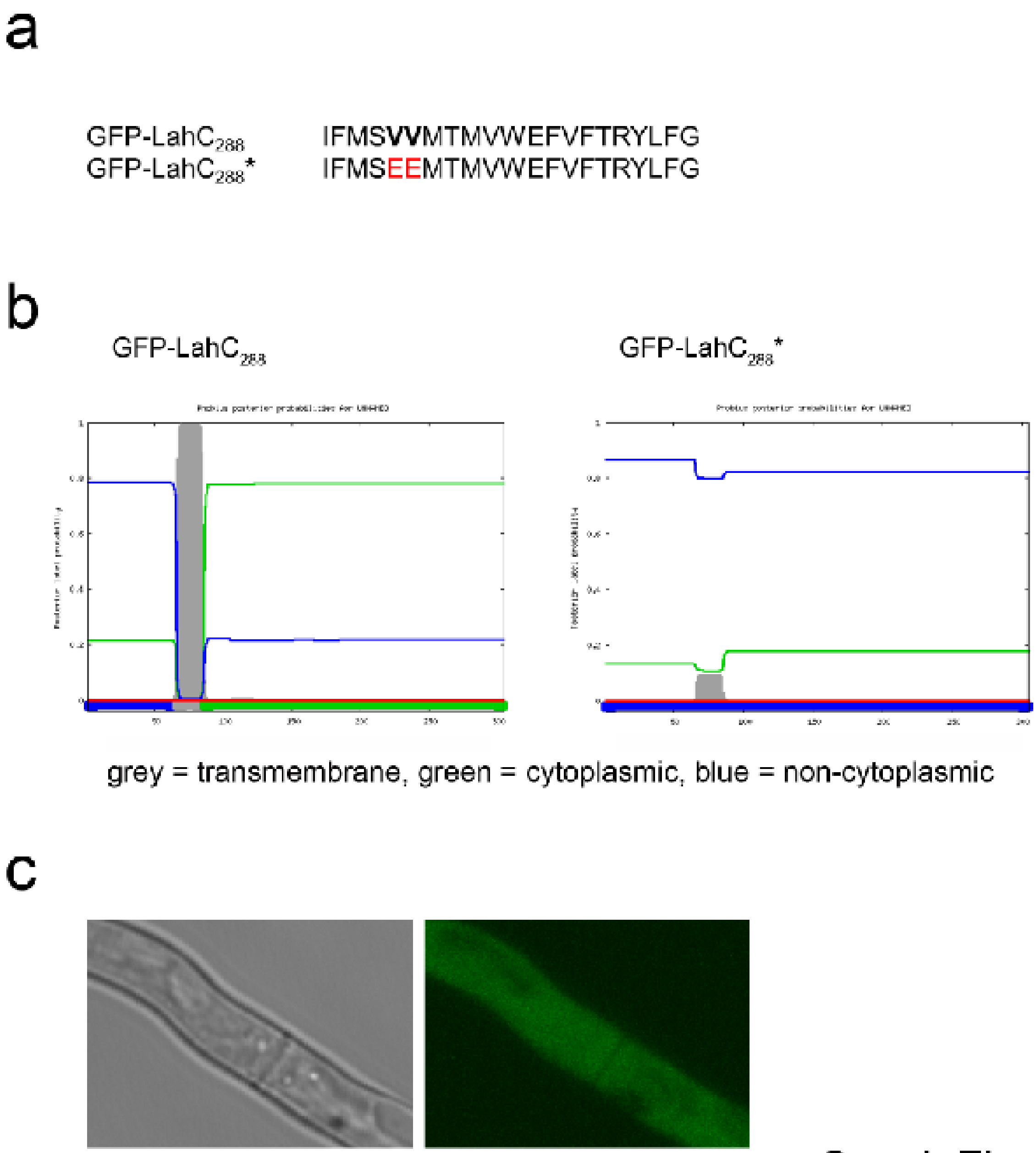

Suppl. Figure 1

Supplementary Figure S1: Generation and characterization of GFP-LahC$_{288}$*. In this construct the predicted transmembrane region was eliminated by point mutations. Panel A shows the amino acid sequences of the membrane-spanning region of GFP-LahC$_{288}$ and the corresponding sequence of GFP-LahC$_{288}$*. Panel B shows PHOBIUS predictions of transmembrane regions for GFP-LahC$_{288}$ and GFP-LahC$_{288}$*. Panel C shows a projection of a stack of confocal images demonstrating the cytoplasmic localization of GFP-LahC$_{288}$*.

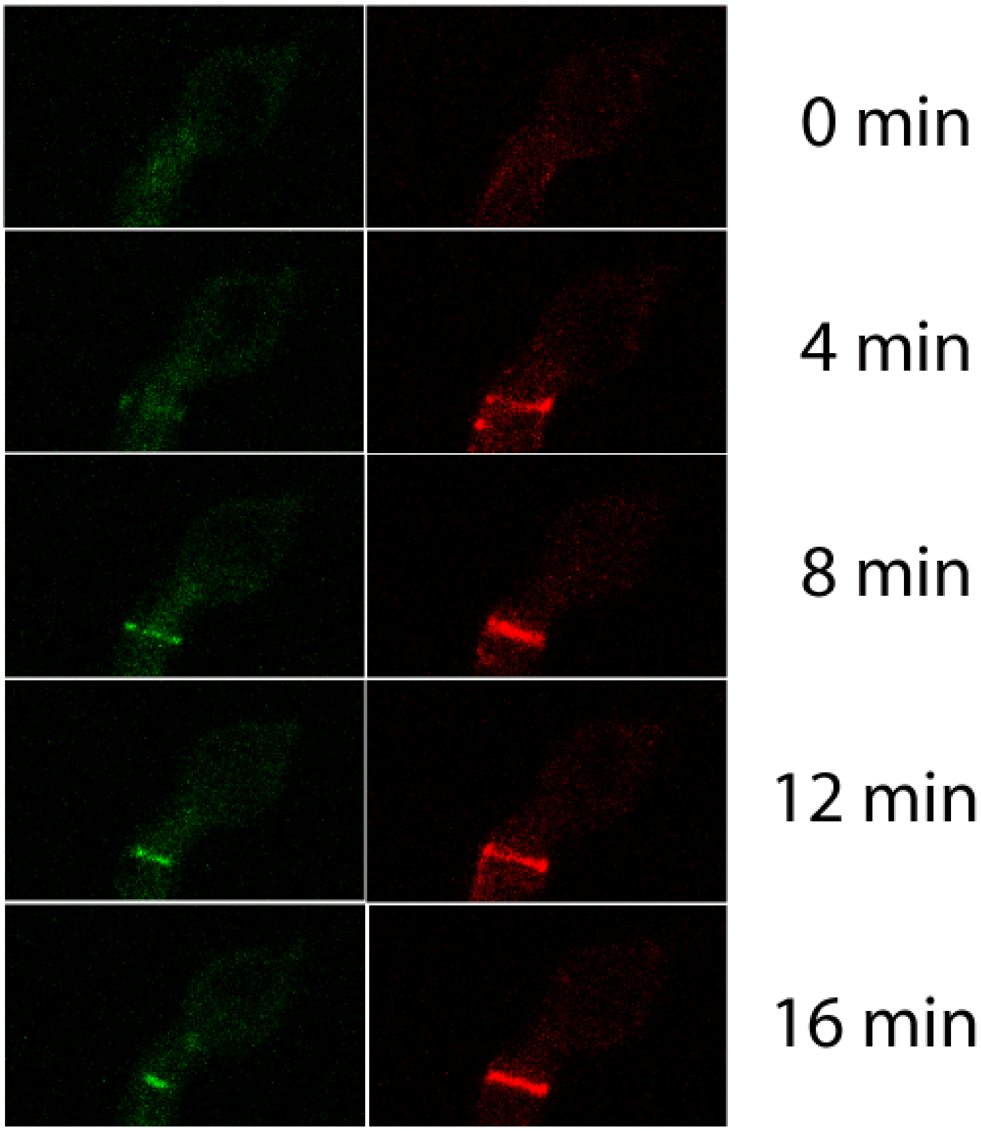

Suppl. Figure 2

Supplementary Figure S2: Localization of GFP-LahC$_{288}$ and the fluorescent actin-binding probe LifeAct-RFP during septum formation. Both proteins were co-expressed in AfS35. Images were taken every 4 min and show projections of stacks of confocal images for GFP-LahC$_{288}$ in green and LifeAct in red.

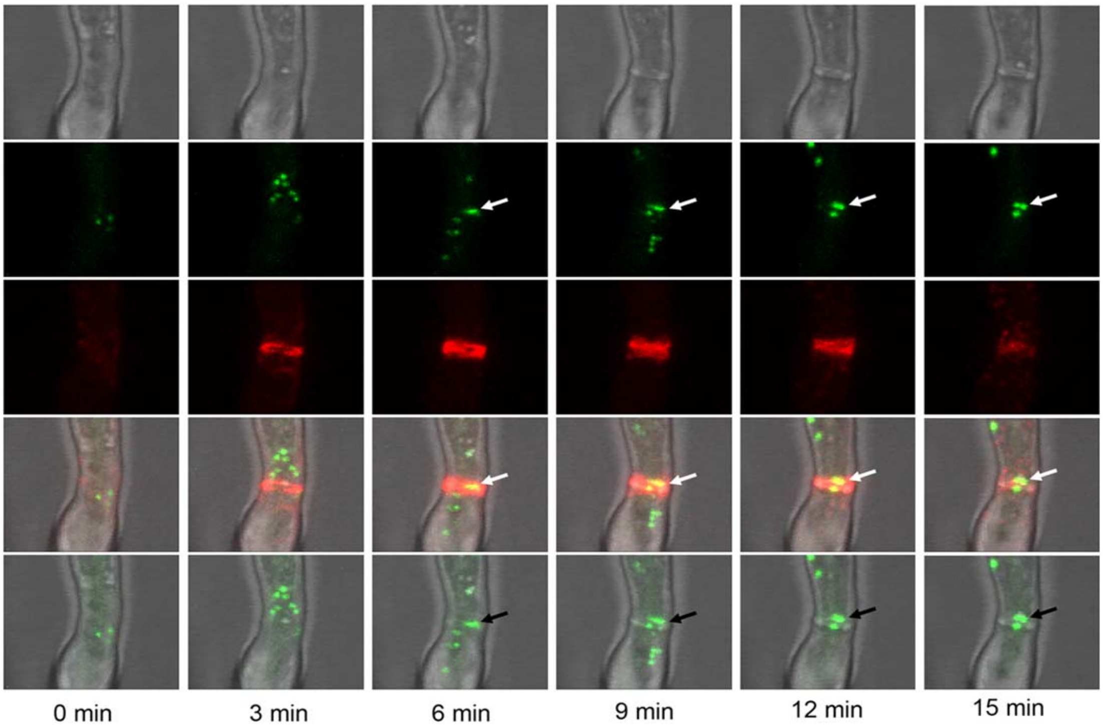

Supplementary Figure S3: Localization of Woronin bodies and filamentous actin during septum formation in wild type hyphae. Live cell imaging of Woronin bodies tagged with LahN-GFP and filamentous actin visualized by LifeAct-RFP in red. Images show projections of stacks of confocal images. Woronin bodies, which are already tethered to the emerging septum, are indicated by arrows.

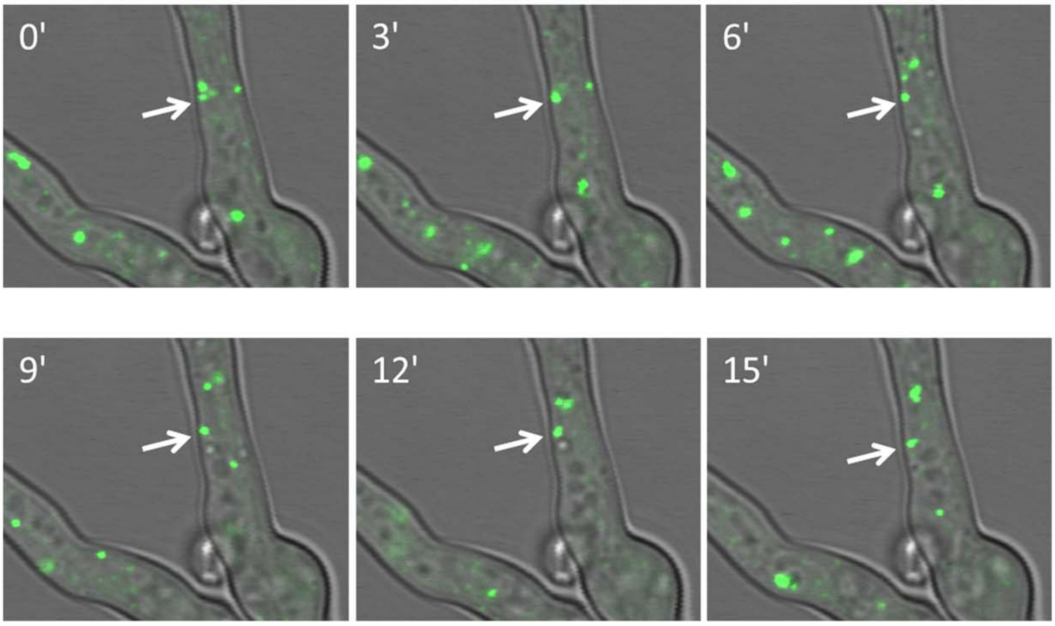

Suppl. Figure 4

Supplementary Figure S4: Live cell analysis of a Δ*rho*4 mutant in which Woronin bodies are tagged by GFP-HexA expression. Images were taken every 3 min. Arrows indicate a Woronin body that remains for 15 min at the lateral cell membrane.

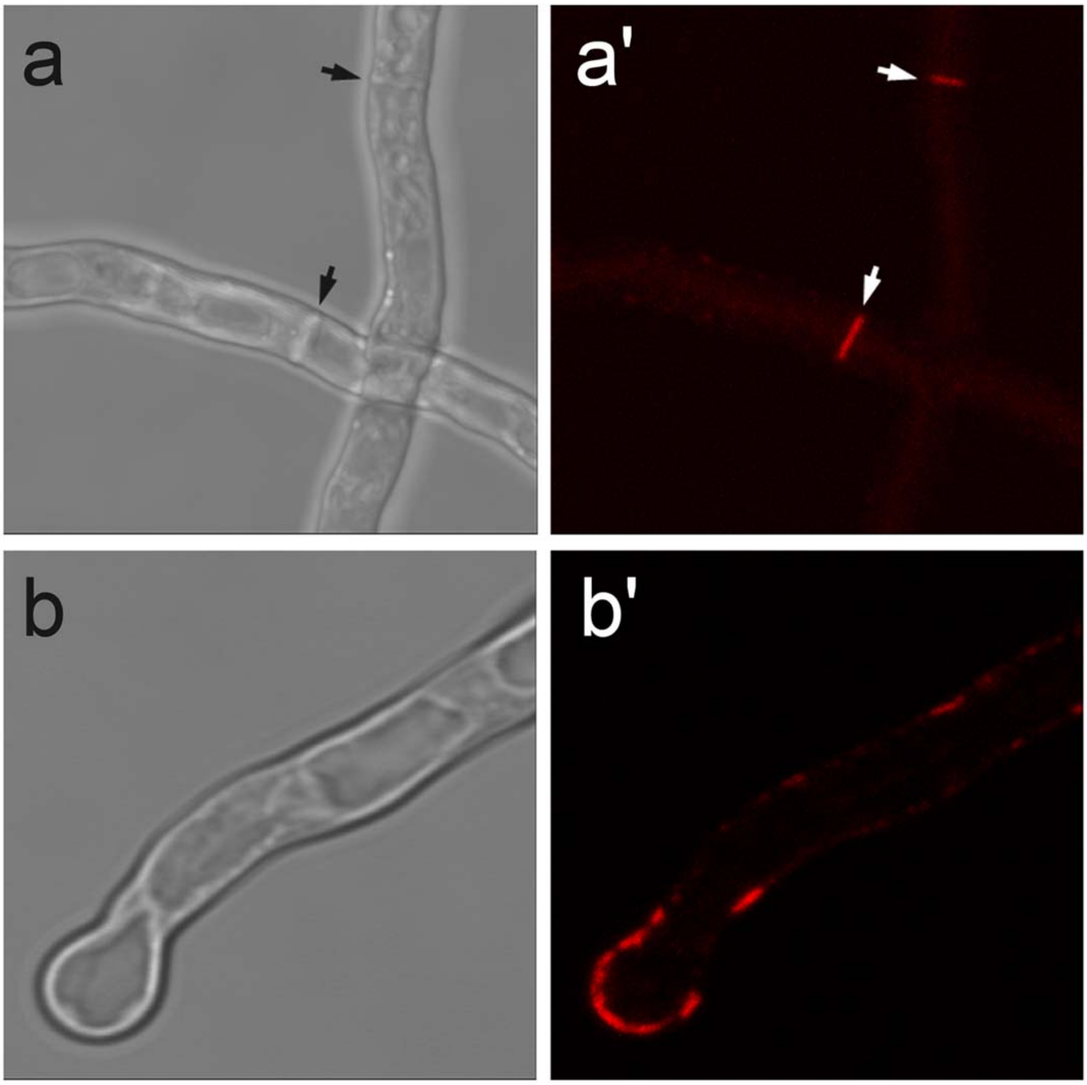

Suppl. Figure 5

Supplementary Figure S5: Localization of the Spa10-RFP fusion protein in wild type hyphae (panels a and a') and hyphae of the Δ*rho4* mutant (panels b and b'). Images represent projections of stacks of confocal images. The positions of septa are indicated by arrows.

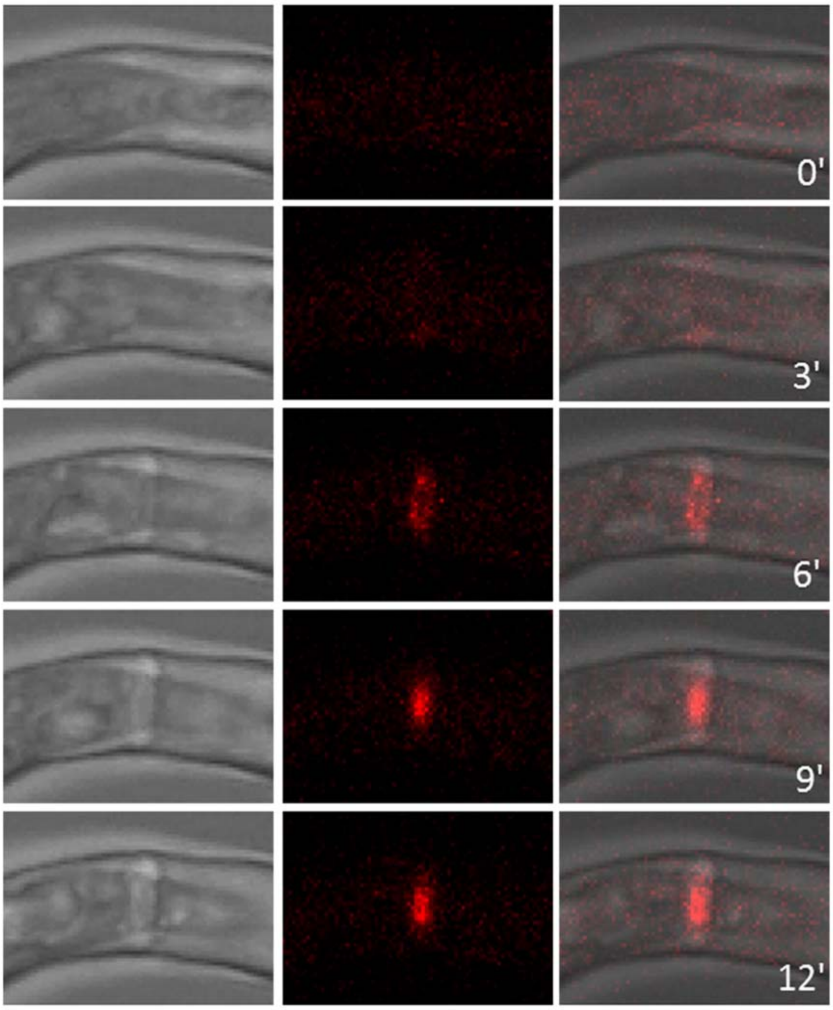

Suppl. Figure 6

Supplementary Figure S6: Recruitment of Spa10-RFP to the emerging septum of *A. fumigatus*. Images show projections of stacks of confocal images and were taken every 3 min.

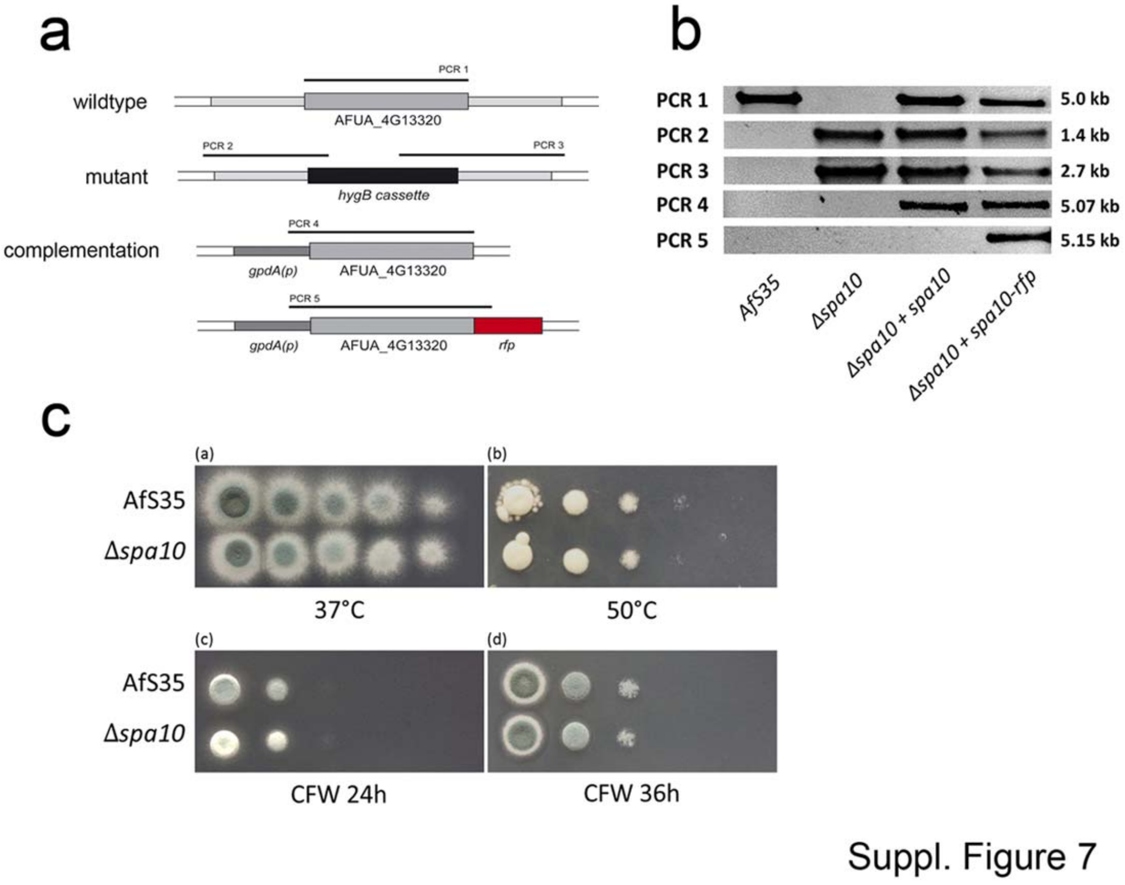

Supplementary Figure S7: Generation and characterization of the Δ*spa*10 mutant. Panel A shows schematic representations of the *spa*10 locus in the wild type and the mutant as well as the constructs used for complementation. The results of analytical PCRs that verify the genetic phenotypes of the different strains are shown in B. The following primer combinations have been used: PCR1 (Spa10-fwd/Spa10-rev), PCR2 (Δspa10-5'-cast/gpdA(p)-3-rev), PCR3 (hph-3-SmaI/Δspa10-3'-cast), PCR4 (gpdA(p)-3-seq-fwd/Spa10-rev) and PCR5 (gpdA(p)-3-seq-fwd/seq-mRFP1-rev). The sequences of these oligonucleotides are summarized in Table 1. Panel C shows drop dilution assays of the mutant and the parental strain AfS35 grown at 37°C and 50°C. In the lower part of this panel, strains were grown on plates containing 25µg/ml Calcofluor white (CFW). Note the slight delay in conidiation of the mutant that is apparent after 24h.

Video 1: Live cell analysis of GFP-LahC288 in the *A. fumigatus* Δ*rho*4 mutant. The images represent projections of stacks of confocal images representing the whole hypha. Images were taken every 3 min. The whole movie represents 108 min.

See attached avi-file.
